# A Young Japanese Patient with Spinocerebellar Ataxia Type 3 Presenting Depressive State with Cenesthopathy and Delusion: a Case Report

**DOI:** 10.1007/s12311-021-01338-4

**Published:** 2021-10-27

**Authors:** Naomichi Okamoto, Atsuko Ikenouchi, Satoru Ide, Yu Hashimoto, Reiji Yoshimura

**Affiliations:** 1 Medical Center for Dementia, University Hospital, University of Occupational and Environmental Health, 1-1 Iseigaoka, Yahatanishi-Ku, Kitakyushu, Fukuoka 8078555 Japan; 2 Department of Psychiatry, University of Occupational and Environmental Health, Kitakyushu, Japan; 3 Department of Radiology, University of Occupational and Environmental Health, Kitakyushu, Japan; 4 Department of Neurology, Neurological Institute Graduate School of Medicine, Kyushu University, Fukuoka, Japan

**Keywords:** Spinocerebellar ataxia type 3, Machado-Joseph disease, Cerebellar cognitive affective syndrome, Psychotic symptoms, Depressive state, Cenesthopathy

## Abstract

Depressive state is a common complication of spinocerebellar ataxia type 3 (SCA3). To the best of our knowledge, cases of SCA3 presenting with cenesthopathy have not been described. Here, we present a case of a severe depressive state with cenesthopathy and delusion in a young Japanese man with SCA3. A 43-year-old Japanese man with SCA3 developed a severe depressive state with associated cenesthopathy and delusion. He was treated with escitalopram (10 mg/day) and olanzapine (2.5 mg/day). Computed tomography showed atrophy of the cerebellum, bilateral superior cerebellar peduncle, and tegmentum of the pons. Single-photon emission computed tomography demonstrated reduced blood flow in the cerebellum, vermis, and brainstem. After 8 weeks, his depressive state and delusion improved; however, his cenesthopathy persisted. We encountered a case of a severe depressive state with cenesthopathy and delusion in a young Japanese man with SCA3. This case supports previous studies that the cerebellum could have a role beyond motor functions.

## Introduction

Spinocerebellar ataxia is a group of autosomal dominant disorders characterized by cerebellar degeneration [1]. Among these, spinocerebellar ataxia type 3 (SCA3), also known as Machado-Joseph disease, is the most common type presenting with progressive cerebellar ataxia and other broad symptoms (e.g., seizures, parkinsonism, dystonia, peripheral neuropathy, psychiatric symptoms, cognitive impairment, sleep disturbances, and olfactory symptoms) [1]. In terms of psychiatric symptoms, most patients with SCA3 experience a depressive state and anxiety [2, 3]. As an attempt to explain the relationship between cerebellar dysfunction and higher-order functional impairment (i.e., executive dysfunction, visuospatial dysfunction, language dysfunction, emotional disorders, and illogical psychotic thoughts), Schmahmann et al. [4] described the cerebellar cognitive affective syndrome (CCAS). They hypothesized that the constellation of deficits could be due to the disruption of the neural circuits that link the prefrontal, posterior parietal, superior temporal, and limbic cortices with the cerebellum [4]. Moreover, cenesthopathy is strongly associated with schizophrenia [5]. Recently, a link between schizophrenia and cerebellar dysfunction has been identified [6], suggesting the possibility of cenesthopathy in patients with SCA3.

Here, we present a case of a severe depressive state with cenesthopathy and delusion in a young Japanese man with SCA3.

## Case Report

A 43-year-old Japanese man with a 15-year history of SCA3 presented to our hospital for a severe depressive state and cenesthopathy. At the age of 28 years, he initially complained of walking instability, which was diagnosed as SCA3 based on clinical symptoms and genetic features. At the age of 30 years, he presented with cervical and truncal dystonia with associated gradual progression of his walking instability. At the age of 42 years, he became completely bedridden.

Sixty days prior to the consult, he presented with cenesthopathy characterized by a feeling that his body was attached to the bed and delusions that he was from the future. Moreover, he complained of anorexia, avolition, restlessness, and psychomotor agitation. Due to the fear of disease progression and the presence of suicidal thoughts, he was referred to our hospital for further assessment and treatment.

The initial psychiatric examination revealed a severe depressive state with a score of 25/52 on the Hamilton Depression Scale-17 (HAMD-17). Moreover, the patient scored 56/126 on the Brief Psychiatric Rating Scale (BPRS). Hematologic and biochemical tests, including thyroid function test, were normal. These findings were consistent with a diagnosis of major depressive disorder with cenesthopathy and delusions associated with SCA3.

We initiated escitalopram (10 mg/day) and olanzapine (2.5 mg/day). Three weeks later, his delusion, depressive state (HAMD-17 score 10/52), and other psychiatric symptoms (BPRS score 40/126) improved. However, his cenesthopathy persisted, which prompted further evaluation.

**Fig. 1:**
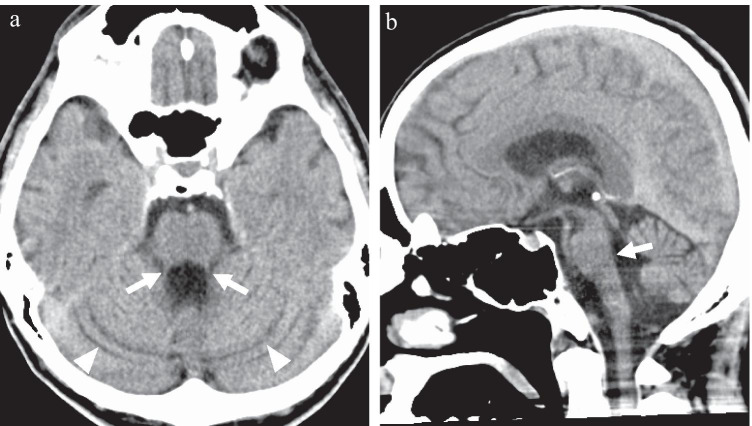
Brain computed tomography (CT). **a** Axial brain CT shows a mild enlargement of the bilateral cerebellar fissures (arrowheads), suggestive of cerebellar atrophy. The image also reveals bilateral superior cerebellar peduncle atrophy (arrow). **b** Sagittal brain CT demonstrates dilatation of the fourth ventricle, suggestive of tegmental atrophy of the pons (arrow)

**Fig. 2:**
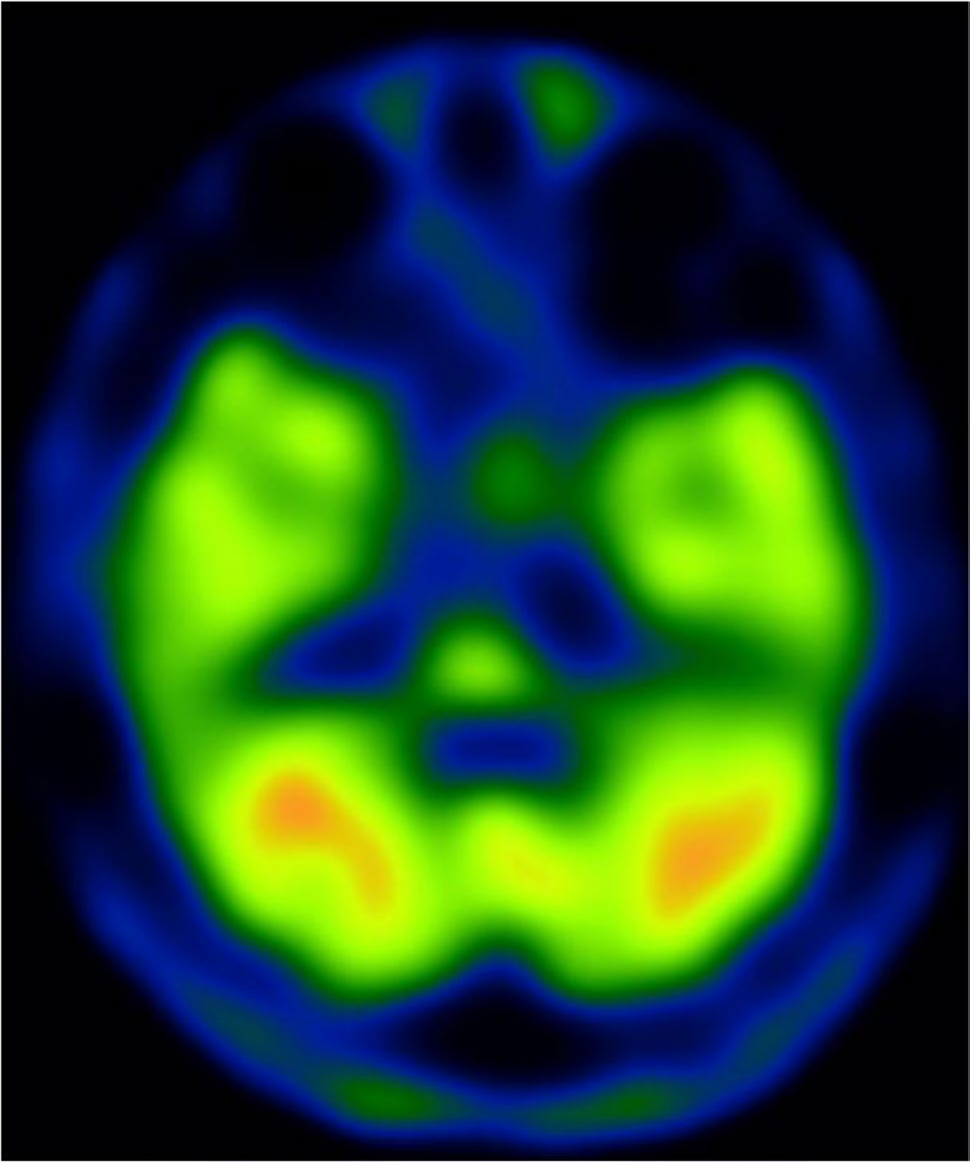
Brain single-photon emission computed tomography (SPECT). Brain SPECT shows decreased cerebral blood flow in the cerebellum, vermis, and brainstem, which are associated with SCA3

Axial computed tomography (CT) of the brain showed mild enlargement of the bilateral cerebellar fissures, suggestive of cerebellar atrophy with associated bilateral superior cerebellar peduncle atrophy (Fig. 1a). Sagittal CT of the brain revealed a dilatation of the fourth ventricle, indicative of tegmental atrophy of the pons (Fig. 1b). Single-photon emission computed tomography (SPECT) of the brain demonstrated decreased cerebral blood flow in the cerebellum, vermis, and brainstem (Fig. 2). No other obvious abnormalities were detected on imaging. These findings were consistent with SCA3.

After 8 weeks of treatment, his depressive state (HAMD-17 score 7/52), delusion, and overall psychiatric symptoms (BPRS score 29/126) improved; however, his cenesthopathy persisted.

## Discussion

A depressive state is a common complication of SCA3. One study found that approximately 33.5% of SCA3 patients had a depressive state [3]. However, to the best of our knowledge, cases of severe depressive state with cenesthopathy in patients with SCA3 have not been described [1].

In a literature search on PubMed, we identified two studies involving patients with spinocerebellar ataxia presenting with psychotic symptoms [1, 7]. A previous study involving 112 patients with spinocerebellar ataxia found that only 5 patients had psychotic symptoms [1]. The mean age of SCA3 patients with psychotic symptoms was 68.4 ± 13.6 years old [1]. A case series by Turk et al. [7] identified patients with spinocerebellar ataxia who presented with delusions, paranoia, and auditory hallucinations. This report described a case of SCA3 with delusion and paranoia in a 30-year-old woman [7]. These findings suggest that this is the first case of SCA3 presenting with cenesthopathy in a young Japanese patient.

Schmahmann et al. [4] described cases of CCAS characterized by executive dysfunction, visuospatial impairment, language dysfunction, emotional disorders, and illogical psychotic thoughts in patients with cerebellar diseases. This suggests the possibility for the presence of neural connections among the cerebellum, non-motor cortical areas, and subcortical areas associated with cognitive and emotional processing [8]. Similar to our case, the SPECT results of a previous study showed that SCA3 patients had significantly lower regional blood flow in the bilateral cerebellum and vermis, compared with healthy subjects [1]. However, no significant differences in terms of regional cerebral blood flow were found between patients with psychotic symptoms and patients without psychotic symptoms [1]. The authors noted that their subjects were relatively old and had atrophy of the basal ganglia. In our case, the patient was young with no obvious abnormalities except for the cerebellum and brainstem on CT or SPECT, ruling out age-related neurodegeneration as an etiology of psychotic symptoms. Moreover, another study implicates psychotic symptoms as an additional non-motor symptom in patients with ataxia due to the connections between the cerebellum and brainstem [4]. This is consistent with our findings of brainstem atrophy.

Cenesthopathy is strongly associated with schizophrenia [5]. While the link between schizophrenia and cerebellar dysfunction has been noted [6], the role of the cerebellum and brainstem in the pathogenesis of cenesthopathy and delusions must be studied further.

Despite the results of our study, there are several limitations identified. First, we did not perform dopamine transporter scans or other tests to assess the degeneration of the basal ganglia. Second, as he was bedridden, cenesthopathy characterized by a feeling that his body was attached to the bed could be understood to some extent.

## Conclusions

We encountered a case of a severe depressive state with cenesthopathy and delusion in a 43-year-old man with SCA3. This report highlights the possibility for patients with SCA3 to present with severe depressive state and unique psychotic symptoms, even in younger patients where age-related neurodegeneration is unlikely. This case supports previous studies that the cerebellum could have a role beyond motor functions.
